# Attribute Feature Classification of English Grammar Entry Base Based on Support Vector Machine Classification Algorithm

**DOI:** 10.1155/2022/2482989

**Published:** 2022-09-13

**Authors:** Wu Yinghua, Meng Shaoxiu, Wang Juan

**Affiliations:** ^1^Langfang Health Vocational College, Langfang, China; ^2^Zhangjiakou University, Zhangjiakou, China

## Abstract

An attribute feature classification method of English grammar vocabulary entry database based on support vector machine classification algorithm is proposed; this method takes news English as the research object and focuses on the classification of attributes and features of the English grammar lexicon database. First, the k-means algorithm is used to cluster the training set, and the one-to-many method is used to train two types of classifiers for the texts that cannot be correctly clustered in each class, that is, the classifiers of the corresponding categories are trained, and then the training set passed through a pair of the classifier generated by multiple SVMs is tested, and the samples that fall in the inseparable area are retrained by a one-to-one method, so as to achieve the purpose of balancing the training samples and reducing the inseparable area. The results show that, compared with the FDAGSVM algorithm, the proposed three multiclass classification algorithms have significantly improved classification speed and classification accuracy, and the macro average accuracy rates are 77.94%, 73.94%, and 72.36%, respectively. While ensuring the classification speed and classification accuracy of the single-label samples, the multiclass classification is realized, and it has high accuracy, recall rate, and value, which better solves the multiclass classification problem and expands the classification capability of the support vector machine. In addition, a comprehensive index based on the SVM classification algorithm is proposed to ensure the specialization of the attribute feature classification.

## 1. Introduction

Since the twenty-first century, global economic integration has become an indisputable fact. Exchanges and cooperation between the international communities are becoming more and more frequent. At the same time, international exchanges and cooperation are always inseparable from the existence of language. As one of the main languages of international communication and cooperation, English is becoming more and more important. Especially with the development of international information society, the demand for English talents is becoming more and more diversified. More and more enterprises begin to pay attention to the selection of compound talents when recruiting English talents, which can help them better carry out international academic exchanges and strengthen international cooperation. As a special language, English is always the most important to master grammar, which is based on a large number of words. For example, news English, because news language itself is a special language specially used to express the news facts. On the one hand, it should have the general commonality of language; on the other hand, it should reflect the personalization of news language. News reports cover a wide range of contents, so news English is very special in terms of economy, objectivity, and readability. First of all, in terms of language style, different publications have different language styles, and different types of articles also have different stylistic characteristics. However, their writing is affected by some common factors, thus forming a common news English feature. First, news publications are mass media with a wide range of readers, and their language must be adapted to the reading level of the vast number of reader's popularity which is one of its major characteristics. Second, the Western press attaches great importance to reading interest. Some journalists call it “the touchstone of news values.” In order to increase the interest of the report, not only the content of the report, but also the language it uses should be adapted to the readers' hobbies and reading habits. Third, thrift is an important means of refining language, and it is also out of the actual need to save space in newspapers and periodicals. In Western society, advertising is lucrative, and newspapers and magazines cherish their space. To report as much as possible in a limited space, news writers must do everything in their power to condense and refine the language. The above three factors, namely, popularity, interest, and frugality, constitute the characteristics of news English in language style. The English words used in news reports are even very different from those used in other occasions, and there will be some differences in grammar, which is also one of the main characteristics of news English grammar and style. Therefore, taking the classification algorithm based on support vector machine as the technical support and news English as the research object, this paper focuses on the attribute feature classification of English grammar entry library.

## 2. Literature Review

**Figure 1:**
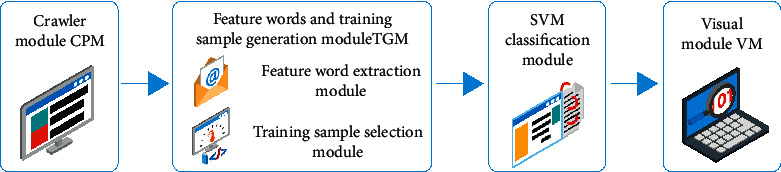
Feature classification of vector machine classification algorithm.

Vijayashree and others say media is a different language. British linguists gave the idea of language change in the 1980s: “Change can be used to refer to existing levels of language [1]. Le et al. describe exchanged terms as “a set of languages with the same taxonomy” [2]. Messina and others said that on this basis, domestic scholars divided language variation into regional variation, social variation, and functional variation [3]. Therefore, news language can be regarded as a form of language variation with the intersection of social variation and functional variation. Ajitha and others said that since the 1990s, English grammar researchers began to study grammar teaching, so as to improve the accuracy of grammar learning [4]. Singer et al. said that the research has proved that the level of grammar knowledge determines the level of a foreign language, and the level of grammar knowledge means the level of a foreign language [5]. Wei et al. indicated that for English-Chinese learners, their literacy skills are weaker, but their literary knowledge level is related to grammatical skills [6]. Some scholars pointed out that if you want to learn grammar well, you should not only have grammar awareness, but also have a lot of practice opportunities. Beigi and others said that a large number of practice opportunities can also play a role in repeatedly cultivating grammar awareness, so as to improve the effect of grammar learning [7]. Therefore, by constantly training grammar topics, learners can establish grammar awareness and get a lot of grammar practice opportunities, so as to improve their English grammar level. Under the traditional offline grammar teaching mode, different schools and teachers have different arrangements for grammar topics. However, Gopi and others said that of course, due to the imbalance of educational resources, learners' learning effects are also different, and students in some areas with low development level will not be able to get better grammar topic training [8]. With the arrival of the information age of education, some online learning platforms begin to appear. The online learning platform turns learning resources from offline to online, and learners can learn anytime, anywhere, regardless of time and geographical location. By the 1990s, with the continuous development of machine learning, data mining, pattern recognition, and other technologies, new machine learning methods have gradually replaced the methods based on knowledge engineering and become the mainstream technology of text classification. Abdi and others said that this classification technology saves a lot of human resources and speeds up the establishment of text classification system [9]. So far, the researchers have proposed a variety of classification algorithms and classification models, including Bayesian algorithm, k-nearest neighbor algorithm, neural network, and support vector machine. These algorithms have been well applied in practice. In the past decade, with the deepening of the research on text classification, a large number of research results have been obtained. Tombak and others said that some scholars used the maximum entropy model for Chinese text classification, and compared and analyzed the classification performance of the classifier based on the maximum entropy model through experiments [10]. Combined with the idea of local linearity and single category, this paper studies the classification of scientific and technological texts. Combined with the idea of local linearity to find the internal support manifold of text samples, and using the idea of single category to determine the interface of positive and negative samples, the classification method of scientific and technological texts has the advantages of controllable classification accuracy of positive and negative samples, good classification effect, and simple parameter estimation. Therefore, an effective way to solve the problem of scientific and technological literature classification is proposed. The feature selection method of text is studied, the Gini index is applied to the feature selection algorithm, and the feature selection evaluation function based on Gini index suitable for text classification is constructed. The attribute feature classification of English grammar entry Library of vector machine classification algorithm is shown in Figure 1.

## 3. Method

### 3.1. Support Vector Machine Classification Algorithm

The support vector machine algorithm is referred to as the SVM (support vector machine) algorithm. The algorithm is based on the VC scale and risk reduction model in the research analysis, combined with the optimization to get the allocation algorithm for allocation decisions. Its basic idea is to find a classification hyperplane and divide the two types of samples into two sides of the hyperplane. It shows many advantages in solving many problems such as nonlinear problems and high-dimensional pattern recognition. It is one of the more practical algorithms in scientific research. Currently, it specializes in tasks such as face recognition, digital writing, text distribution, and data retrieval [11].

#### 3.1.1. Two Types of Linearly Separable SVMS

Linear separability means that a classification hyperplane can be found to correctly separate two classes. The given text training set is shown

(1) $$\begin{array}{c} T=\left\{\left(x_{1},y_{1}\right),\left(x_{2},y_{2}\right),\ldots ,\left(x_{k},y_{k}\right)\right\}\in {\left(R^{n}\times Y\right)}^{k}, \end{array}$$

where *x*_*i*_ ∈ *R*^*n*^, *y*_*i*_ ∈ *Y*={1, −1}, *i*=1,…, *k*, there is *w* ∈ *R*^*n*^, *b* ∈ *R* an d positive number *δ*, so that the set satisfies the following formula :

(2) $$\begin{array}{c} \left\{\begin{array}{c} \left(w\cdot x_{i}\right)+b\geq \delta ,\\ \left(w\cdot x_{i}\right)+b\leq -\delta , \end{array}\right.\begin{array}{c} y_{i}=1\text{,}\\ y_{i}=-1\;i=1,\ldots ,k\text{.} \end{array} \end{array}$$

The text training set *t* is said to be linearly separable.

**Figure 2:**
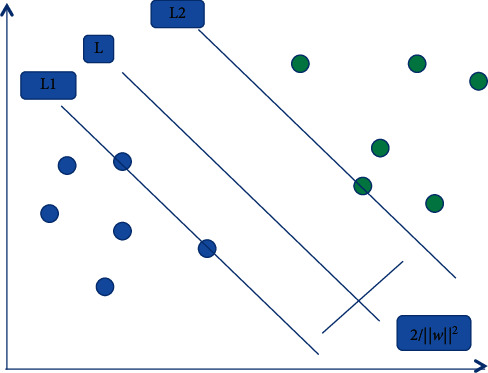
Optimal classification line.

In the case of linearly separable, the basic concept of SVM is shown in Figure 2. The black and white dots in the figure are the two types of classified text, *l* is the best maximum distribution line of the two types, L1 and L2 are the models closest to and equal to the distribution line, and the distance between the two lines is called classification interval. The support vector machine algorithm is to find the best distribution line, that is, the distribution line that can not only separate the two groups, but also make the distribution orderly. (2) is then further normalized as in

(3) $$\begin{array}{c} y_{i}\left[\left(w\cdot x_{i}\right)+b\right]\geq 1\;i=1,\ldots ,k. \end{array}$$

At this time, L1 and L2 meet the equation, respectively, as shown in the following formula:

(4) $$\begin{array}{c} y_{i}\left[\left(w\cdot x_{i}\right)+b\right]=1\text{,}\\ y_{i}\left[\left(w\cdot x_{i}\right)+b\right]=-1\text{.} \end{array}$$

If the classification interval distance is 2/‖*w*‖^2^ and the optimal classification surface satisfies formula (3) and 2/‖*w*‖^2^ is maximized, the text on L1 and L2 is called support vector. If 2/‖*w*‖^2^ is maximized, it is equivalent to 1/2‖*w*‖^2^ is minimized. In this way, the problem is transformed into a nonlinear programming problem, as shown in formulas (5) and (6):

(5) $$\begin{array}{c} \min_{w,b}\frac{1}{2}{\left\|w\right\|}^{2}\text{,} \end{array}$$

(6) $$\begin{array}{c} s.t\;y_{i}\left[\left(w\cdot x_{i}\right)+b\right]\geq 1\;i=1,2,\ldots ,k\text{.} \end{array}$$

According to the optimization theory, there is a unique minimum solution for the above problem. The Lagrange function is as shown in formula (6):

(7) $$\begin{array}{c} L=\frac{1}{2}{\left\|w\right\|}^{2}+\overset{k}{\underset{i=1}{\sum }}\alpha_{i}\left[1-y_{i}\left(w\cdot x_{i}+b\right)\right]{\;\alpha }_{i}\geq 0 \end{array}$$

where *α*_i_ is the Lagrange multiplier. Take the derivative of w and *b* in the above formula and make the derivative zero, that is, as shown in formula (7):

(8) $$\begin{array}{c} \frac{\partial L}{\partial w}=w-\overset{l}{\underset{i=1}{\sum }}\alpha_{i}y_{i}x_{i}=0\Rightarrow w=\overset{k}{\underset{i=1}{\sum }}\alpha_{i}y_{i}x_{i}\\ \frac{\partial L}{\partial w}=\overset{k}{\underset{i=1}{\sum }}\alpha_{i}y_{i}=0. \end{array}$$

Bring formulas (8) into (7) and transform the optimal problem into its dual problem, as shown in the following formula:

(9) $$\begin{array}{c} \max\;w\left(\alpha \right)=\overset{k}{\underset{i=1}{\sum }}\alpha_{i}-\frac{1}{2}\overset{k}{\underset{i,j=1}{\sum }}\alpha_{i}\alpha_{j}y_{i}y_{j}\left(x_{i}\cdot x_{j}\right)\\ \overset{k}{\underset{i=1}{\sum }}y_{i}\alpha_{i}=0\\ \alpha_{i}\geq 0,i=1,\ldots ,k\text{.} \end{array}$$

By solving the above dual problem, the optimal distribution area can be obtained, and the distribution decision function is

(10) $$\begin{array}{c} f\left(x\right)=sgn\left(w*x+b^{*}\right)=sgn\left(\overset{k}{\underset{i=1}{\sum }}\alpha_{i}^{*}y_{i}x_{i}\cdot x+b^{*}\right). \end{array}$$

When judging the category of the text to be tested, it can be classified by formula (10).

#### 3.1.2. Two Kinds of Linear Nonseparable SVM

Linear indivisibility means that the categories cannot be completely separated by an optimal classification plane. At this time, the limiting conditions in formula (7) can be appropriately relaxed and a relaxation factor *ξ*_i_ can be introduced, and formula (3) becomes as shown in formula (11):

(11) $$\begin{array}{c} y_{i}\left[\left(w\cdot x_{i}\right)+b\right]\geq 1-\xi_{i}\;i=1,\ldots ,k\text{.} \end{array}$$

When *ξ*_i_ is large enough, all texts will be included. In order to limit *ξ*_i_, the penalty factor C is introduced, and the limiting conditions are added, as shown in the following formula:

(12) $$\begin{array}{c} c\overset{k}{\underset{i=1}{\sum }}\xi_{i}\;i=1,\ldots ,k\;c>0\text{.} \end{array}$$

At this time, the problem is transformed into another optimization problem, as shown in the following formulas:

(13) $$\begin{array}{c} \min_{w,b}\frac{1}{2}{\left\|w\right\|}^{2}+c\overset{k}{\underset{i=1}{\sum }}\xi_{i}\text{,} \end{array}$$

(14) $$\begin{array}{c} s.t\;y_{i}\left[\left(w\cdot x_{i}\right)+b\right]\geq 1-\xi_{i}\text{,} \end{array}$$

(15) $$\begin{array}{c} \xi_{i}\geq 0\;i=1,\ldots \text{,}k. \end{array}$$

It is transformed into its dual problem, as shown in the following formula:

(16) $$\begin{array}{c} \max\;\;w\left(\alpha \right)=\overset{k}{\underset{i=1}{\sum }}\alpha_{i}-\frac{1}{2}\overset{k}{\underset{i,j=1}{\sum }}\alpha_{i}\alpha_{j}y_{i}y_{j}\left(x_{i}\cdot x_{j}\right),\\ \overset{k}{\underset{i=1}{\sum }}y_{i}\alpha_{i}=0,\\ 0\leq \alpha_{i}\leq c,i=1,\ldots ,k. \end{array}$$

The transformed discriminant function is similar to the linear separable time. The transformed SVM method to solve the linear nonseparable problem can prevent the poor classification effect caused by noise. High-dimensional mapping is the second core component of support vector machines. We know that the biggest feature of linear classifiers is simplicity. To put it bluntly, it is “one rib.” When faced with nonlinear classification problems, it does not know how to work around it, so we need to help it to unblock it, just like solving the problem of Logistic regression, high-dimensional mapping is what we are looking for. SVM methods based on two-class distribution problems are generally described above.

### 3.2. Neural Network Algorithms

**Figure 3:**
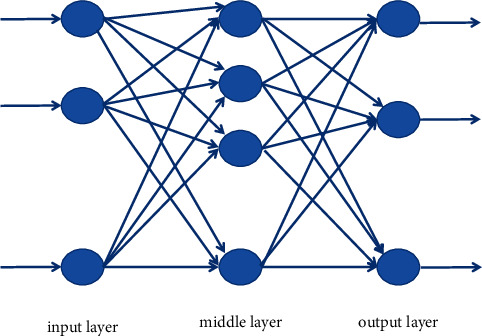
Classification diagram of the neural network.

A neural network is an algorithm that behaves like an animal neural network and is used to average the distribution of information. It is a network of neurons. Each neuron has only one connection, but the output connection can connect as many connections as needed, and these connections output the same signal, that is, the signal of the corresponding neuron, but the size of the signal is different and it varies with the number of branches [12]. According to the difference of network structure and learning algorithm, artificial neural network can be divided into multilayer perceptron, self-organizing map, and Hopfield network. The following takes BP neural network as an example to illustrate the application of neural network in text distribution. BP neural network is a multilayer perceptron network researched according to the error reproduction algorithm. It consists of an inlet layer, an outlet layer, and at least one intermediate layer. As shown in Figure 3, each neuron in the input layer is responsible for receiving input data and sending it to each neuron in the intermediate layer; the intermediate process is an internal process responsible for data transmission. According to the needs of data transmission capability, the intermediate process can be designed as a single layer or layer by layer, and further process the data transmitted from the intermediate layer to each cell in the publishing process to complete the expansion process. The learning process is done once, and the release of information is completed by the process of external release. When the actual output does not match the expected release, the error recovery stage is entered [13]. The error goes through the release process, changing the weight of each layer in the way of gradient error descent, returning to the middle layer, and entering layer by layer. The process of repeating data for forward propagation and forced back propagation is the process of continuously adjusting the weights of each layer, and is also the process of neural network learning and development training. This process continues until the output error decreases to an acceptable level or the set learning time is reached as shown in Figure 3.

Because the neural network simulates the human nervous system, it enhances self-organization and self-learning ability and has good robustness. He has made great achievements in understanding the structure and distribution of text.

### 3.3. Decision Tree Algorithm

**Figure 4:**
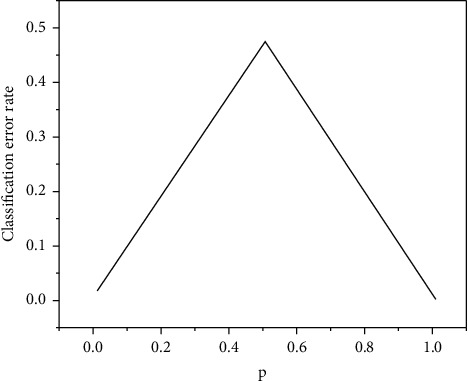
Curve of Gini coefficient and half entropy.

Decision tree is a multilevel classification algorithm based on tree structure, which consists of root nodes, nonleaf nodes, and leaf nodes [14, 15]. Each nonpage represents a test of one or more behaviors, and the page node represents a category. One way from the base point of the leaf to the leaf is the distribution law of similar objects, which can easily turn the tree decision into a distribution law. It is an intuitive classification method. The process of determining the tree can be divided into two steps. The first step is decision tree design: the process of creating a decision tree by training a model. The second stage is the tree-building pruning technique: tree-building pruning is the process of completing the tree-building process of the previous stage. Before designing with data from test data, it is important to check the rules, a decision tree is a complex tree generated by fully considering all the data points, and there may be overfitting. The more complex the decision tree, the higher the degree of overfitting and prune branches that affect the prediction accuracy. In the process of designing the tree, the key is to select the material characteristics of the internal nodes, that is, which feature item is used in nonleaf nodes to make decisions on samples. The decision-making process is closer to human thinking, so the model is easier to interpret; the model can be described more clearly with graphics; it is fast; it can handle continuous and discrete data; does not require any domain knowledge and parametric assumptions; and suitable for high-dimensional data. Information gain is usually used to calculate the feature item value of each node. According to the value of the feature item, the feature item with the largest value is selected as the test attribute of the current node each time, so as to reduce the mixing degree of different categories in each subset [16]. At present, there are a lot of research on decision tree derivation, decision tree attribute selection, and decision tree scalability, and many algorithms and systems based on decision tree have been developed, including ID3, ID4, C45, SLIQ, and Sprint. You can compare the expression of Gini coefficient with the expression of entropy model. Is quadratic operation much simpler than logarithm? In particular, the calculation of class II classification is simpler. But how big is the error corresponding to Gini coefficient compared with the measurement method of entropy model? For the Gini curve of entropy sum, as shown in Figure 4.

### 3.4. Support Vector Machine and Class Classification Algorithm

**Figure 5:**
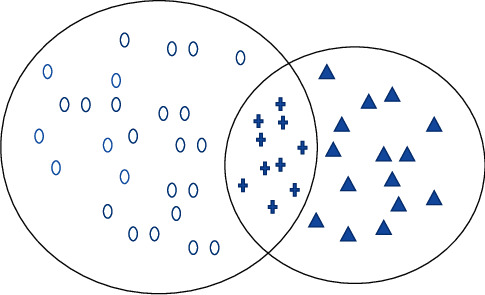
Schematic diagram of multiclass classification of hypersphere support vector machine.

Hyperspheric SVMs are only used to solve the class distribution problems. Only one model is required for training, and the training speed is faster, it can be extended to many classification problems. Multiple hypersphere support vector machines are constructed to achieve the purpose of multiclass classification, that is, each class of samples is trained separately, and a classifier is established for each class, as shown in Figure 5.

Experimental dataset 1, select more than 3 papers from 685 papers in 5 categories for experimental analysis. 445 texts serve as standard training material and 240 additional texts serve as standardized tests, as shown in Table 1.

**Figure 6:**
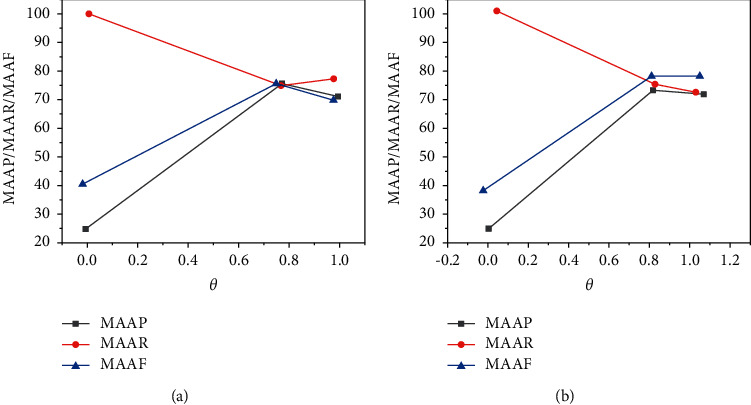
(a) Changes of macro average accuracy, macro average recall, and macro average in the calculation process. (b) Changes in macro average accuracy, macro average recall, and macro average in the linear accounting method.

For the calculation process, the macro mean and the macro mean calculated by the difference of variables are shown in Figure 6(a).

For the linear accounting method, the changes of macro average accuracy, macro average recall, and macro average under different values are shown in Figure 6(b).

**Figure 7:**
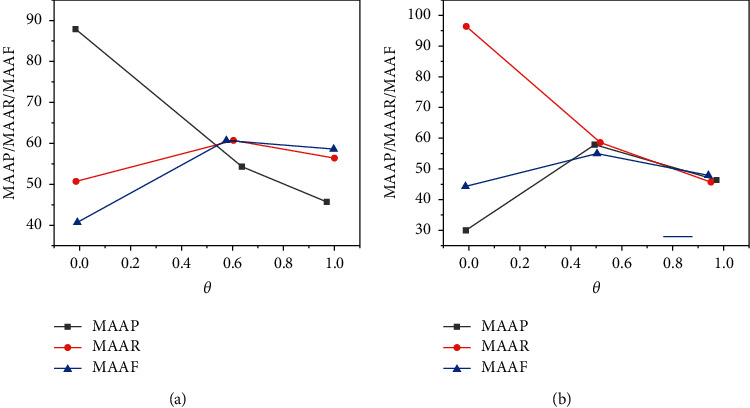
(a) Changes of macro average accuracy, macro average recall, and macro average under different values according to algorithm differences. (b) Changes of macro average accuracy, macro average recall, and macro average under different values according to algorithm differences.

The macro mean, macro mean return, and macro mean variables according to algorithm differences are shown in Figure 7(a).

The changes of macro average accuracy, macro average recall, and macro average under different values of the algorithm are shown in Figure 7(b).

In order to compare the performance of the algorithm, the three multicategory classification algorithms proposed are experimentally analyzed on the same dataset, as shown in Table 2, and the specific parameters are shown in Table 3.

The experimental results show that, compared with the FDAGSVM algorithm, the proposed three multiclass classification algorithms have significantly improved classification speed and classification accuracy, and the macro average accuracy rates are 77.94%, 73.94%, and 72.36%, respectively. While ensuring the classification speed and classification accuracy of single-label samples, it realizes multiclass classification, and has higher accuracy, recall rate, and value, better solves the problem of multiclass classification, and expands the classification ability of support vector machines.

## 4. Attribute Feature Classification of English Grammar Lexicon Based on Support Vector Machine Classification Algorithm

### 4.1. English Grammar Entry Attribute Feature Classification Performance Index

The quality of entry classification algorithm is mainly measured by the classification results of classifier. The classification results of entry are mainly evaluated from three aspects: computational complexity, simplicity, and effectiveness of description. Computational complexity is divided into space and time complexity. If classified according to the classification steps, the computational complexity can be divided into training and classification computational complexity [17, 18]. The brevity of description is the brevity of algorithm description, which can also be understood as the difficulty of algorithm understanding. Effectiveness is the ability of a classifier to classify correctly. Among these three aspects, the effectiveness of classifiers is the most important, so the evaluation of effectiveness is the main content of classifier evaluation [19]. In the evaluation of effectiveness, precision (*P*) and recall (*R*) are the most commonly used indicators in Chinese and English entry classification. Precision refers to the proportion of entry that really conforms to the classification intention after classifying the test documents, which reflects the accuracy of the system search results. Recall rate refers to the ratio of the number of documents that really meet the retrieval intention in the retrieved result document set to all the document sets that meet the retrieval intention, which reflects the completeness of the system retrieval. The formulas of precision and recall can be expressed as follows:

The duplicate check rate is

(17) $$\begin{array}{c} p=\frac{cp_{i}}{k_{i}}\text{.} \end{array}$$

Recall is

(18) $$\begin{array}{c} R=\frac{cp_{i}}{c}\text{,} \end{array}$$

where *c* is the number of samples actually belonging to *c*_i_ class, *k*_i_ is the number of samples predicted as ci by the classifier, and *cp*_*i*_ is the number of samples correctly classified as ci class. The higher the value of precision and recall, the better the performance of the classifier. However, these two standards restrain each other, that is, simply increasing the precision will lead to the reduction of recall, and simply increasing the recall will lead to the reduction of precision. Therefore, in order to avoid a certain index being too low, a good classification algorithm needs to make some trade-offs between the two [20]. Therefore, *F*1 value is used to describe the comprehensive effect of precision and recall. The formula of *F*1 value of each type is

(19) $$\begin{array}{c} F1=\frac{2P_{i}R_{i}}{\left(R_{i}+P\right)}\text{,} \end{array}$$

where *P*_i_ is the precision rate of class *i* and *R*_i_ is the recall rate of class *i*. The overall *F*1 value is the average value of various *F*1 values. It can be seen from the formula that *F*1 represents the balance relationship between *P* and *R*. *F*1 can be large only when both *P* and *R* are large. Therefore, *F*1 value comprehensively reflects the integrity of the classifier.

### 4.2. English Grammar Entry Attribute Feature Classification Linguistics Index

For the classification of the attribute features of English grammar entry, in addition to grasping the performance indicators, we should also consider some indicators of professional linguistic knowledge of English entry. Only in this way can the classification of entry be distinguished from the classification of natural language and highlight the unique features of English grammar entry.

#### 4.2.1. New Words

Journalistic English is a masterpiece of modern English, such as the solemn statement of the president, the relaxed conversation of the people, and the jargon of various disciplines. News reporting style often uses news reporting idioms, which have repeatedly appeared in newspapers and magazines within a specific period of time and have become the “jargon” of the press [21, 22]. Due to social progress and the development of science and technology, new phenomena, new ideas, and new trends continue to appear. The original words and languages cannot carry or transmit new ideas and new information, so people begin to make new words. The unique word use tendency of these new words and news language often appears in the mass media, especially in newspapers and periodicals. Journalistic English is a very useful key for linguists to explore the development trend of modern English. The frequent emergence of new words mainly has the following three situations in terms of composition and form of expression: adding new meaning to old words means that a word has obtained a new category of word meaning, and the writing form is still the original old words, but the word meaning has been changed through extension, metaphor, and other ways. With the wide use of these words, they gradually infiltrate into the language of daily life [23]. For example, on October 4, 1958, the former Soviet Union launched the world's first man-made satellite, opening up a new era of human exploration of the universe. English news reports are spelled “Sputnik I satellite” in Russian letters. After that, a series of new words with “- nik” as suffix to express “with - Characteristics” appeared in English news media [24, 25]. The common ones are: beatnik (beat generation refers to a group of young people who are dissatisfied with social reality and despise traditional ideas in the United States after World War II); Computernik (computer expert); Folknik (folk song fan); Cinemanic (movie fan); Peacenik (peace loving person); jazznik (jazz fan); and Jobnik (workaholic). Now, it is a foregone conclusion that this form of new words composed of noun ten “- nik”, most of which are included in the dictionaries because of their wide use. In English news reports, reporters often skillfully add prefixes or suffixes to an old word to form a new word as an “expedient” to make up for the shortcomings of the old and limited meaning of the original English words and difficult to meet the needs of real life. There are many new words of this kind, which is also a remarkable feature that news English is different from the other English styles [26]. News English has an obvious innovative spirit in the use of words, which also has a great impact on the development and change of the whole English. Its composition is very simple. It connects common phrases together, omits some syllables, and combines them into one. Some loanwords have been completely English due to their frequent use. For example, To San Francisco fengshui guru Steven Post, who has seen interest grow from a “trickle to a tor-rent,” the assumption that people are affected by their surroundings is common sense. (Newsweek) “Fengshui” directly borrows Chinese characters and is synonymous with it. As English newspapers and magazines often report news events in other countries, it is inevitable to borrow words that have the current characteristics and strong cultural color of the field. More and more Chinglish words are accepted by foreigners, showing China's increasing international influence. The occasional use of foreign words with exotic colors can not only attract the interest and attention of native English readers, but also give native English readers a sense of cultural intimacy and identity. “Meaning, the most important part of language, is often associated with changes in life and social thinking. Rapid changes in life, including changes in social accuracy and changes in science and technology, often lead to many changes in language.” In particular, nonnative speakers need to query the meaning of figurative language more consciously, even more than native speakers. Newspapers often use satirical and harmonious reports to increase the humor and effect of news language, especially in headlines. News English has a wide audience, and their educational level is very different, which requires reporters to try to use vivid words that most people can understand. The most common in English newspapers is to use capital names to change countries or governments, as well as use product names, design features, and methods to represent governments and organizations to keep the media alive and dynamic, which is an important part of English news. In a word, understanding the lexical features and lexical tendencies of English is more conducive to the scientific classification of English grammatical items from the technical level.

#### 4.2.2. Common Metaphors and Synonyms

News English has a wide audience, and their educational level is very different, which requires reporters to try to use vivid words that most people can understand. The most common in English newspapers is to use capital names to change countries or governments, as well as use product names, design features, and methods to represent governments and organizations to keep the media alive and dynamic, which is an important part of English news. Example: World leaders set up the White House to listen. Leaders of many countries are vying for the White House presidency.

#### 4.2.3. The Phenomenon of Euphemism

Another important aspect of English-language media is the frequent use of euphemisms. Euphemism is vague and deceptive. Western political life is the fertile soil for euphemism. In daily life, euphemism is for “taboo” or “Politeness,” while in political life, euphemism is for the purpose of “disguise”.

#### 4.2.4. Literary Allusions Words

There are many literary allusions and words in newspapers and periodicals. For example: A droopy dollar, caused by the growing U.S. trade deficit, may be the Achilles heel in what remains a surprisingly robust domestic expansion (U.S. News &World Report). “The Achilles heel” is the heel of Achilles (Greek mythology), which is a metaphor for fatal weakness.

## 5. Conclusion

Support vector machine is a new machine learning method based on statistical learning theory. It uses structural risk minimization criteria to design learning machine, which can better solve nonlinear, high-dimensional, and local minimum problems. Entry classification is a research hotspot in the field of information retrieval and data mining, which is closely related to the development of machine learning. On the one hand, the new method of machine learning promotes the rapid development of entry classification, and this method puts forward many challenging issues. This paper studies the main methods of multiclass support vector machine in English grammar entry attribute feature classification, such as one-to-one support vector machine and one-to-many support vector machine, and proposes a support vector machine classification algorithm to solve the sample imbalance and inseparable region problems in entry classification. This method firstly uses k-means algorithm to cluster the training item set, selects the entry with clustering errors in a certain class, and other classes as positive and negative items, and trains the classifier of this class with one-to-many support vector machine, which solves the problem of unbalanced number of training entry in the process of training the classifier of one-to-many SVM. Then, the classifiers generated by the one-to-many support vector machines are used to test various types of training sets, and the entry falling into the indivisible region are retrained by the one-to-one support vector machine method, thereby reducing the indivisible area of the one-to-many support vector machines. Through a series of experiments, it is proved that the support vector machine classification algorithm is feasible for the attribute feature classification of English grammar entry 

## Figures and Tables

**Table 1:** Training corpus and test corpus.

Dataset	A	B	C	D	E
Training set size	168	44	44	79	204
Test set size	84	23	23	40	101

**Table 2:** Parameter settings of four algorithms.

Parameter	FDAGSVM	1-a-1MC	1-a-rMC	HSMC
C	50	100	20	—
*τ*	0.05	0.005	0.005	0.01
*ε*	—	0.01	0.01	—
*θ*	0.8	0.8	0.6	—
*ν*	—	—	—	0.6

**Table 3:** Comparison of macro average accuracy, macro average recall, and macro average *F*1 values of four algorithms.

Algorithm	Macro average accuracy (%)	Macro average calling rate (%)	Macro average *F*1 (%)
FDAGSVM	60.84	59.24	60.31
1-a-1 MC	77.94	76.32	75.85
1-a-rMC	73.94	76.04	72.93
HSMC	72.36	72.64	71.49

## Data Availability

The datasets generated and analyzed during the present study are available from the corresponding author upon request.
